# The use of whole exome sequencing and murine patient derived xenografts as a method of chemosensitivity testing in sarcoma

**DOI:** 10.1186/s13569-018-0090-1

**Published:** 2018-03-08

**Authors:** Nicholas Calvert, Jiansha Wu, Sophie Sneddon, Jennifer Woodhouse, Richard Carey-Smith, David Wood, Evan Ingley

**Affiliations:** 1 Department of Orthopaedic Surgery, Sir Charles Gairdner Hospital, Nedlands, WA 6009 Australia; 2 Murdoch University, Murdoch, WA 6150 Australia; 3 Harry Perkins Institute of Medical Research, The University of Western Australia, 6 Verdun Street, Nedlands, WA 6009 Australia; 4 Hollywood Functional Rehabilitation Centre, 117 Stirling Hwy, Nedlands, WA 6009 Australia

**Keywords:** Sarcoma, Chemosensitivity, Whole exome sequencing

## Abstract

**Background:**

Soft tissue and bone sarcoma represent a broad spectrum of different pathology and genetic variance. Current chemotherapy regimens are derived from randomised trials and represent empirical treatment. Chemosensitivity testing and whole exome sequencing (WES) may offer personalized chemotherapy treatment based on genetic mutations.

**Methods:**

A pilot, prospective, non-randomised control experimental study was conducted. Twelve patients with metastatic bone or soft tissue sarcoma that had failed first line chemotherapy treatment were enrolled for this study. Human tissue taken at surgical biopsy under general anaesthetic was divided between two arms of the trial. Subsections of the tumour were used for WES and the remainder was implanted subcutaneously in immunodeficient mice (PDX). Results of WES were analysed using a bioinformatics pipeline to identify mutations conferring susceptibility to kinase inhibitors and common chemotherapeutic agents. PDX models exhibiting successful growth underwent WES of the tumour and subsequent chemosensitivity testing.

**Results:**

WES was successful in all 12 patients, with successful establishment PDX tumours models in seven patients. WES identified potential actionable therapeutics in all patients. Significant variation in predicted therapeutics was demonstrated between three PDX samples and their matched tumour samples.

**Conclusion:**

Analysis of WES of fresh tumour specimens via a bioinformatics pipeline may identify potential actionable chemotherapy agents. Further research into this field may lead to the development of personalized cancer therapy for sarcoma.

**Electronic supplementary material:**

The online version of this article (10.1186/s13569-018-0090-1) contains supplementary material, which is available to authorized users.

## Background

The mainstay of systemic cancer therapies has been empirically derived cytotoxic combination protocols based on histological appearance, organ of origin, and tumour staging. This approach has proven effective in lymphomas and other germ cell tumours, but their efficacy has been less in bone and soft-tissue sarcoma due to their heterogeneity [1]. For patients with chemotherapy resistant sarcoma, the significant toxicity of these agents result in morbidity and mortality without therapeutic benefit. This has led to an interest in developing ex vivo chemosensitivity testing to predict drug response prior to patient administration.

Recent advances have been made in the utilization of genomic data to facilitate development of targeted therapeutic agents. An example of this is the identification of key genes and signaling pathways in gastrointestinal stromal tumours (GIST). Identification of oncogene mutations in *KIT*, *PDGRFα* and *BRAF* have led to the development of selective kinase inhibitors to target them [2–4].

The whole human genome contains approximately 3 × 10^9^ base pairs, containing coding (exons) and non-coding (introns) regions [5]. Less than ~ 10% of the genome is characterized and the clinical implications of mutations throughout the genome are poorly understood [6]. It is estimated that 85% of disease-causing mutations are found in the coding region of the genome, the exome [7, 8]. As such, limiting the analysis to exome sequencing rather than whole genome sequencing provides the most cost-effective method and the greatest possibility of identifying clinically relevant mutations.

The implantation of patient derived tumours into immunodeficient mice is a recently developed technique to provide a more representative microenvironment for measuring tumour response to chemotherapy agents. Reliable mouse models that recapitulate the biological profiles of sarcomas have been developed as a research tool in sarcomas [9] and indeed one such model led to the description of a possible cell of origin of synovial sarcoma [10]. These xenografts provide a more complete model of the biological behaviour and metastatic potential of tumours than cell line studies [11, 12].

Tyrosine kinases play an important role in the modulation of cell signalling pathways. Their role in oncogenesis of several forms of cancer was recently discovered and arises from genetic mutation that causes dysregulation of these pathways to stimulate a variety of biologic pathways, including angiogenesis and cell growth. Tyrosine kinase inhibitors competitively inhibit ATP binding or impede activation/activity of these kinases, thus leading to pathway deactivation [13].

The use of these drugs in sarcoma has been limited in comparison to other malignancies. In a recent review [14], it was shown that the response rate of soft tissue sarcomas to a variety of kinase inhibitors in phase 2 and phase 3 trials remained well below 10%. However, it should be noted that these trials tended to group together different tumour types. As previously mentioned, sarcoma has a high tendency for heterogeneity between types and subtypes, hence these broad studies may not provide a representative view on the efficacy of this drug class. By identifying gene mutations targetable by tyrosine kinase inhibitors in individual sarcoma samples it may be possible to tailor therapies and improve response rates.

Herein, we describe a comparison of whole exome analysis of germline, tumour, and murine patient derived xenografts (PDX), with implementation of a bioinformatics pipeline for chemosensitivity prediction in 12 patients with bone and soft tissue sarcoma.

## Methods

A prospective case series study was conducted from March 2016 to March 2017. Institutional approval to conduct the trial was obtained from the Human Ethics Committee. Twelve adult patients with bone or soft tissue sarcoma who had failed to respond to standard chemotherapy, or newly diagnosed local recurrence of bone or soft tissue sarcoma following chemotherapy, were enrolled in the trial. Under-age patients, or those with a cognitive impairment preventing them from providing informed consent were excluded from the trial. Informed consent was obtained by an investigator.

Following informed consent, samples of the tumour were taken at the time of surgical biopsy under general or local anaesthesia.

### Generation of patient-derived xenografts (PDX)

Subsections of the tumour sample were divided between whole exome sequencing (WES) and implantation subcutaneously in immunodeficient mice. Patient blood samples were sent for WES for germline exome identification. DNA was extracted from tumour and blood using a Blood and Cell Culture DNA mini kit according the manufacturer’s instructions (Qiagen, Hilden, Germany). DNA was quantitated using a NanoDrop (ThermoFisher Scientific, Waltham, MA) and integrity checked by agarose electrophoresis and ethidium bromide staining. Tumour tissue was sliced into approximately 2 mm^3^ fragments, mixed with extracellular matrix (Matrigel, at 1 mg/mL) (Corning, Tewksbury, MA) and implanted subcutaneously via an 18G needle on the right flank of 6–10 week-old female NSG (NOD.Cg-*Prkdc*^*scid*^ *IL2Rγ*^*tm1Wjl*^/SzJ) mice (Jackson Laboratory, Bar Harbor, ME). PDX models with successful establishment of tumours were subsequently biopsied with sections of the tumour sent for WES. All PDX tumour biopsies performed for exome sequencing were performed from zero passage mice to minimise the amount of model acquired mutations, which have been demonstrated to increase with each passage [15].

### Exome sequencing and data processing

Samples for WES were prepared using an Agilent SureSelect Target Enrichment Kit and libraries were sequenced via 100 bp PE using an Illumina HiSeq 4000 sequencer (Macrogen, Seoul, Republic of Korea). With 100× read depth for germline DNA, and 200× read depth for primary tumour and PDX DNA.

### Application of the IMPACT pipeline

Sequence data were then analysed using a modified version of the Integrating Molecular Profiles with Actionable Therapeutics (IMPACT) pipeline developed at the University of Colorado [16]. All analysis parameters were as per those outlined by Hintzsche et al. [16].

Further functional analysis of the gene mutations identified as a potential kinase inhibitor target was performed to investigate whether targeting these kinases had known effects on tumour biology. Known molecular pathways were explored using WebGestalt [17] and identification of the site of these mutations on the kinase proteins were explored using the UniProt Knowledge Database [18].

The actionable therapeutics identified for each PDX sample was compared with it’s matched list of tumour derived actionable therapeutics using Fisher’s exact test of independence for assessment of statistical significance.

## Results

**Fig. 1:**
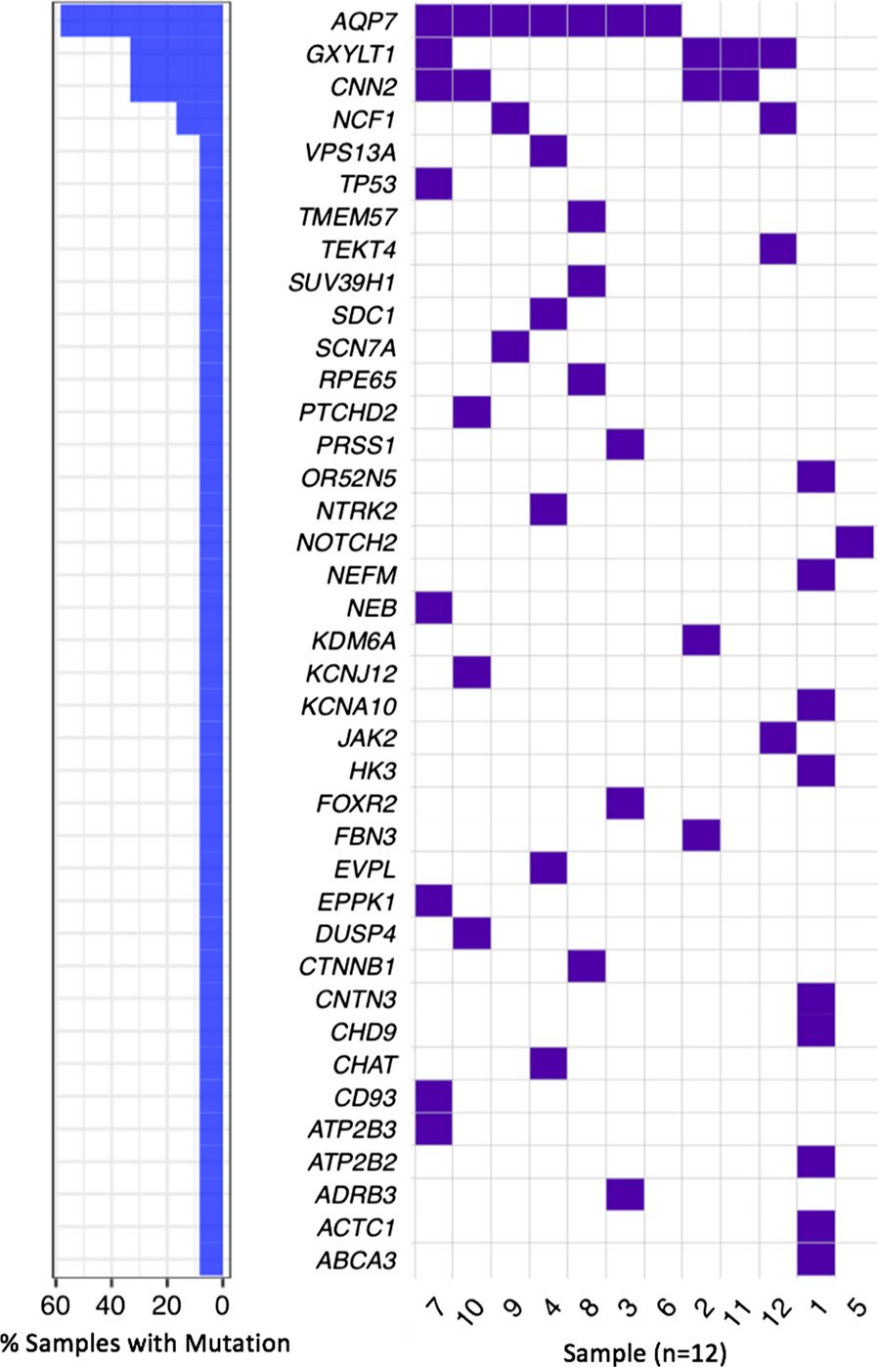
Waterfall plot of single nucleotide variants predicted to be deleterious or likely deleterious

Establishment of tumours in PDX models was successful in seven patients. Patient demographics are outlined in Table 1. Figure 1 lists identified single nucleotides variants that were predicted to be deleterious by six algorithms (SIFT, Polyphen2, MutationTaster, FATHMM, CADD, GERP) [19–24] or by two algorithms if the variant is listed in COSMIC [25].

**Table 1:** Patient demographics

Patients (n)	12
Male	6
Female	6
Median age (range)	49 (23–68)
Type of sarcoma (n)	
Osteosarcoma	2
Undifferentiated pleomorphic sarcoma	2
Leiomyosarcoma	2
Synovial sarcoma	1
Ewing’s sarcoma	1
Alveolar rhabdomyosarcoma	1
Chordoma	1
Metastatic angiosarcoma	1
Dedifferentiated liposarcoma	1

### Sequencing metrics

Sequencing metrics are outlined in Table 2. Whole exome sequencing was performed to a median depth of 142. A median of 122,945,876 reads were generated for each sample. A median of 475 somatic mutations per sample were identified for the tumour samples, with a median of 34,442 for the PDX.

**Table 2:** Sequencing metrics

Diagnosis	Depth	Reads (M)	% on-target reads	Somatic mutations	Synonymous	Non-synonymous	INDEL	Frame-shift	Stop gain/loss
Osteosarcoma									
Germline	88.1	75	89.1						
Tumour	153.2	134	81.5	479	144	290	12	24	9
PDX	282.7	141	53.9	96,393	79,080	15,354	440	1334	139
Dedifferentiated liposarcoma									
Germline	74.7	71	89.8						
Tumour	148.5	123	79.4	458	154	250	14	28	12
Leiomyosarcoma									
Germline	94.9	778	79.0						
Tumour	145.7	116	81.1	384	118	218	15	26	7
Osteosarcoma									
Germline	72.1	613	74.7						
Tumour	175.2	148	79.0	575	179	331	18	30	17
PDX	142.1	123	80.2	288,522	232,825	48,320	1496	5307	477
Synovial sarcoma									
Germline	71.5	784	58.3						
Tumour	159.6	134	79.5	471	169	245	13	32	12
Ewing’s sarcoma									
Germline	65.8	730	58.4						
Tumour	122.7	128	61.0	529	191	262	21	45	10
PDX	141.7	125	75.5	7089	3399	3416	115	105	53
Undifferentiated pleomorphic sarcoma									
Germline	77.1	677	75.4						
Tumour	139.6	132	72.5	522	202	271	15	26	7
PDX	168	137	79.4	118,641	97,602	18,574	568	1651	207
Alveolar rhabdo-myosarcoma									
Germline	83.1	727	86.6						
Tumour	152.6	132	86.3	591	187	325	25	39	12
PDX	187.3	159	76.7	24,851	12,266	10,617	121	331	117
Leiomyosarcoma									
Germline	98.8	880	76.7						
Tumour	166	147	80.2	368	112	220	5	20	11
PDX	159.8	134	77.9	34,442	28,019	5701	183	470	67
Chordoma									
Germline	70.7	617	77.5						
Tumour	145.8	141	78.9	440	147	247	7	31	8
Metastatic angiosarcoma									
Germline	72	632	76.1						
Tumour	202.2	176	78.2	489	177	249	17	36	10
PDX	147	121	77.7	10,376	7892	2238	42	148	52
Undifferentiated pleomorphic sarcoma									
Germline	78.6	692	82.4						
Tumour	146	119	82.6	405	147	210	16	25	7

### Direct tumour analysis

Potential kinase inhibitor targets were identified in six of the twelve samples. These samples were of osteosarcoma, Ewing’s sarcoma, undifferentiated pleomorphic sarcoma, alveolar rhabdomyosarcoma, and leiomyosarcoma. The results of this analysis are outlined in Table 3.

**Table 3:** Predicted kinase inhibitors

Patient	Diagnosis	Database	Gene	Variant	Therapeutic
Pt1	Osteosarcoma	NCI match clinical trials	KIT	E142Q	Sunitinib
		DSigDB FDA approved kinase inhibitors	KIT	E142Q	Imatinib, Sorafenib, Dasatanib, Sunitinib, Nilotinib, Pazopanib, Axitinib, Cabozantinib
Pt6	Ewing’s sarcoma	DSigDB FDA approved kinase inhibitors	ALK	C928fs	Crizotinib, Ceritinib
Pt7	Undifferentiated pleomorphic sarcoma	DSigDB FDA approved kinase inhibitors	ABL2	I471fs	Dasatinib
Pt8	Alveolar rhabdomyosarcoma	DSigDB FDA approved kinase inhibitors	NTRK1	G18E	Imatinib
Pt9	Leiomyosarcoma	DSigDB FDA approved kinase inhibitors	ALK	C928fs, F921L	Crizotinib, Ceritinib
Pt12	Undifferentiated pleomorphic sarcoma	DSigDB FDA approved kinase inhibitors	JAK2	S593F	Ruxolitinib

Analysis using the bioinformatics pipeline successfully identified potential actionable therapeutics in all the twelve samples. An example of the generated list of potential actionable therapeutics is outlined in Table 4. The results for all patients are listed in Additional file 1.

**Table 4:** Predicted actionable therapeutics for patient 1

Drug	Targets hit	Potential targets	*P* value (hypergeometric test)
Letrozole	1	5	< 0.001
Fludarabine	1	6	0.001
Sunitinib	1	9	0.001
Gemcitabine	1	11	0.002
Imatinib	1	10	0.002
Clofarabine	1	12	0.002
Regorafenib	1	18	0.005
Cytarabine	1	18	0.005
*Doxorubicin*	1	104	0.133
*Cisplatin*	–	–	–
*Methotrexate*	–	–	–
*Ifosfamide*	–	–	–

Diagnosis was of osteosarcoma. Therapeutics that the patient had received are highlighted in italics

One sample of osteosarcoma demonstrated a mutation of the KIT gene. This gene is a proto-oncogene responsible for the encoding of c-kit, and plays a role in cell survival and proliferation. The mutation identified (E142Q) lies in the extracellular domain and may alter the structure of the receptor. The same mutation of the ALK gene (C928fs) was demonstrated in a sample of Ewing’s sarcoma and leiomyosarcoma. The exact action of this ALK mutation on oncogenesis is still unclear, however it has been implicated in numerous malignancies including non-small cell lung cancer [26]. The identified frameshift mutation is located extracellularly before the kinase domain, and may result in a decoy receptor being produced. A mutation of ABL2 was demonstrated in a patient with undifferentiated pleomorphic sarcoma. This gene has been implicated in numerous solid organ tumours [27] and plays a role in cell growth and survival. The identified frameshift (I471fs) lies within the protein kinase domain, and may alter receptor function. A second patient with the same tumour demonstrated a JAK2 mutation, a kinase with important roles in cell growth and development. The observed mutation (S593F) was present in the second protein kinase domain, which is thought to be responsible for catalytic activity. Finally, a mutation of NTRK1 was identified in a sample of alveolar rhabdomyosarcoma. Gene fusions with this gene are potentially oncogenic via up regulation of the TRKA protein. The mutation demonstrated (G18E) lies outside of the functional domains and as such selective inhibition would be not appear to be beneficial.

In four patients, actionable therapeutics were identified that are already in use as treatment for the corresponding sarcoma: Methotrexate was identified for an osteosarcoma patient as well as a synovial sarcoma patient; Doxorubicin was identified for an Ewing’s sarcoma patient and Vincristine was identified for an alveolar rhabdomysarcoma patient.

### PDX actionable therapeutics comparison

Establishment of PDX tumour lineages was successful for seven patients. Failure of tumour establishment was defined as lack of observable tumour growth within 24 weeks.

All seven PDX tumours underwent WES from which potential actionable therapeutics were identified. Samples from three patients were assessed to be statistically significant different in terms of actionable therapeutics identified in direct tumour and PDX analysis (Table 5). The primary tumours for these three samples were osteosarcoma, undifferentiated pleomorphic sarcoma, and leiomyosarcoma.

**Table 5:** Comparison of potential actionable therapeutics between direct tumour analysis and PDX analysis

Diagnosis	PDX-therapeutics identified (n)	Novel in comparison to tumour (n)	Tumour-therapeutics identified (n)	P-value
Osteosarcoma	6	6	9	1.000
Osteosarcoma	24	22	11	*0.019*
Ewing’s sarcoma	16	15	13	0.161
Undifferentiated pleomorphic sarcoma	19	11	29	*< 0.001*
Alveolar rhabdomyosarcoma	6	5	3	0.219
Leiomyosarcoma	9	4	6	*0.025*
Metastatic angiosarcoma	11	11	10	1.000

Each of the seven PDX samples are listed with their primary diagnosis. P-values were calculated using Fisher’s exact test of independence

Italic values indicate significance of P-value (p < 0.05)

## Discussion

This study outlines a method for the use of WES in combination with the IMPACT bioinformatics pipeline to generate a list of potentially actionable chemotherapeutics in bone and soft tissue sarcoma.

Analysis of the WES data with the bioinformatics pipeline found tyrosine kinase inhibitor targets for half of the patients. Kinase targets found in the analysis were for osteosarcoma (*KIT*), Ewing’s sarcoma (*ALK*), undifferentiated pleomorphic sarcoma (*JAK2, ABL2*), alveolar rhabdomyosarcoma (*NTRK1*), and leiomyosarcoma (*ALK*). Of these targets, two have been described previously in the literature for their respective malignancy. Two separate studies [28, 29] have described the presence of *ALK* mutations in Ewing’s sarcoma and have hypothesized that this mutation may be targetable with Crizotinib. Over expression of the KIT gene and resultant increased levels, has previously been identified as a potential therapeutic target in paediatric osteosarcoma [30].

As this is a non-validated method this trial did not aim to alter patient drug treatment. Indeed, due to advance stage of disease in which the biopsies were performed, the majority of patients were deceased at the time of results becoming available. Of note, one patient with osteosarcoma elected to undergo treatment with the predicted tyrosine kinase inhibitor whilst in the terminal stages of their disease. A temporary clinical response was demonstrated with a reduction in size of his recurrent tumour. Whist this is far from conclusive, such a result provides some promise that guided treatment may be feasible.

A list of potentially actionable therapeutics was generated for all twelve fresh tumour specimens. Four patients had received chemotherapy agents that were predicted to be actionable, and in none of these were more than one of their current agents predicted to be actionable. The interpretation of this result is difficult, as enrolled patients had already undergone chemotherapy treatment. Darwinian selection pressure in response to chemotherapy exposure may have resulted in the tumours already developing resistance to previous chemotherapy agents. Identifying whether these predicted agents truly are effective is difficult. As this method of drug prediction is not validated we were unable to utilise these results in chemotherapeutic selection. Due to the heterogeneity of sarcoma the data on non-traditional chemotherapy regimes is scarce. Therefore, a larger trial is required with biopsy performed prior to commencement of chemotherapy, and subsequent tracking of chemotherapy response. This would clarify whether there is correlation between predicted drugs and clinical course.

Of the genes predicted to be deleterious four were present in more than one sample. A mutation of AQP7 was present seven samples. This gene encodes for Aquaporin 7, and there is some early evidence that it may play a role in tumour cell function [31]. Four demonstrated mutations in GXYLT1 and four had mutations in CNN2. GXYLT1 is involved in the Notch protein pathway, it’s potential role in these malignancies is unclear. CNN2 is known to play a role in smooth muscle contraction and cell adhesion. A sample of alveolar rhabdomyosarcoma and a sample of undifferentiated pleomorphic sarcoma both demonstrated a mutation in NCF1, mutation of which can be associated with chronic granulomatous disease.

Results of pipeline analysis of PDX samples to identify tyrosine kinase inhibitors indicated varied results in comparison to fresh tumour samples. Of the seven PDX models, five had targetable mutations identified in fresh tumour samples with only three of these remaining present in the PDX analysis. Analysis of all seven PDX samples demonstrated the presence of novel targetable gene mutations in comparison to fresh tumour. The most marked of this was the osteosarcoma sample from patient 4, which demonstrated no actionable kinase inhibitor targets in the tumour WES and 35 novel actionable kinase targets with 227 novel variants in the PDX WES.

Actionable chemotherapeutics were successfully predicted in all seven PDX samples. Pipeline analysis identified novel chemotherapeutics in all samples in comparison to fresh tumour results. Samples from three patients were found to be significantly different statistically in terms of actionable therapeutics identified in direct tumour and PDX analysis. These three samples were for osteosarcoma, undifferentiated pleomorphic sarcoma, and leiomyosarcoma.

Of the twelve patients enrolled in this trial two had osteosarcoma, two had leiomyosarcoma, and two had undifferentiated pleomorphic sarcoma. The two osteosarcomas demonstrated different mutation, kinase inhibitor, and actionable therapeutic profiles. The leiomyosarcoma samples had different drug profiles but both demonstrated a mutation in AQP7. Likewise, the undifferentiated pleomorphic sarcomas had different drug profiles but a mutation of the gene GXYLT1 was present in both.

The results obtained in this study raise several interesting questions. Despite poor earlier evidence for the use of tyrosine kinase inhibitors in sarcoma, the results of the pipeline analysis may suggest that they could play a role if selected individually for patients. It is possible that the genetic mutations that confer a susceptibility to these agents occurs sporadically throughout the sarcoma subtypes. Therefore, careful selection may provide a response in a subset of patients. Secondly, it is interesting to note that very few of the patient’s received chemotherapeutics that were predicted to be actionable by pipeline analysis. An intriguing finding is that pipeline analysis of a chordoma sample indicated the presence of 17 potential actionable therapeutics. Except in rare cases of aggressive chordoma, this sarcoma has traditionally been regarded as having a poor response to chemotherapy [32].

Several limitations are inherent in the design of this study. As this is a pilot investigation, the small number and heterogeneity of the population studied prevents any definitive conclusions to be made. As such, this study is intended to generate hypotheses for further studies and establish a protocol for a larger trial. In comparison to Stebbing et al. [9] a lower rate of successful graft incorporation in the PDX models was seen, 58% in comparison to 75%. The reason for this is unclear, as the patient population and technique were similar, and may relate to the smaller sample size in this trial. Although a list of potentially actionable therapeutics was generated for each patient, due to the small sample size it is not possible to meaningfully compare the predicted therapeutics to the observed clinical response of the tumour to the received chemotherapeutics. A larger patient population would be required to establish any significant correlation between the results. Lastly, the heterogeneity of the patient tumour type acts as a limitation in this study, a limitation that can be noticed in a significant proportion of the sarcoma literature. However, it could be argued that due to the rarity and vast heterogeneity of these tumours amongst type and subtype, which is becoming increasingly apparent with genetic analysis, it may not be possible to apply the traditional study design to this malignancy successfully. Therefore, the use of “n of 1” trial designs and similar methods may have a significant role to play.

It should be noted that this is a pilot study and further research into this area is required. This is especially important when considering cost and infrastructure required for large scale multi-centre trials. Continued research into the use of WES and drug prediction using bioinformatics pipelines, such as IMPACT, for sarcoma are strongly encouraged. Larger numbers are required to permit meaningful correlation of predicted drug responses and observed clinical response. As this technique is not clinically validated, such a trial would provide support for the development of a multi-centre prospective trial in which chemotherapy selection is guided by chemosensitivity pipeline results.

## Conclusion

This study provides support for the development of a large-scale trial utilising WES and the IMPACT bioinformatics pipeline to determine potential actionable chemotherapy agents and tyrosine kinase inhibitors. With further advances it is possible that drug prediction via WES may provide clinicians with the ability to deliver personalized therapy to patients with sarcoma and other malignancies.

## Additional file

**Additional file 1.** The full list of potential actionable therapeutics for all twelve patients.
