# Supplementary material for: The use of whole exome sequencing and murine patient derived xenografts as a method of chemosensitivity testing in sarcoma
Source: Clin Sarcoma Res. 2018 Mar 8;8:4. doi: 10.1186/s13569-018-0090-1 (PMC5842605; doi:10.1186/s13569-018-0090-1)
Supplement: Supplementary file 1 — Additional file 1. The full list of potential actionable therapeutics for all twelve patients. [file 13569_2018_90_MOESM1_ESM.docx]

**SUPPLEMENT 1: Direct Tumour Analysis – Potential Actionable Therapeutics**

**Patient 1**

Diagnosis: Osteosarcoma

Therapeutics received: Methotrexate, cisplatin, doxorubicin, ifosfamide

| **Drug** | **Targets Hit** | **Potential Targets** | **P-value (hypergeometric test)** |
| --- | --- | --- | --- |
| Letrozole | 1 | 5 | <0.001 |
| Fludarabine | 1 | 6 | 0.001 |
| Sunitinib | 1 | 9 | 0.001 |
| Gemcitabine | 1 | 11 | 0.002 |
| Imatinib | 1 | 10 | 0.002 |
| Clofarabine | 1 | 12 | 0.002 |
| Regorafenib | 1 | 18 | 0.005 |
| Cytarabine | 1 | 18 | 0.005 |
| **Doxorubicin** | 1 | 104 | 0.133 |
| **Cisplatin** | - | - | - |
| **Methotrexate** | - | - | - |
| **Ifosfamide** | - | - | - |

**Patient 2**

Diagnosis: Dedifferentiated Liposarcoma

Therapeutics received: Mesna, Doxorubicin, Ifosfamide, Dacarbazine

| **Drug** | **Targets Hit** | **Potential Targets** | **P-value (hypergeometric test)** |
| --- | --- | --- | --- |
| Paclitaxel | 1 | 35 | 0.023 |
| **Doxorubicin** | - | - | - |
| **Mesna** | - | - | - |
| **Dacarbazine** | - | - | - |
| **Ifosfamide** | - | - | - |

**Patient 3**

Diagnosis: Leiomyosarcoma

Therapeutics received: Mesna, Doxorubicin, Ifosfamide, Dacarbazine

| **Drug** | **Targets Hit** | **Potential Targets** | **P-value (hypergeometric test)** |
| --- | --- | --- | --- |
| Fludarabine | 1 | 6 | 0.001 |
| Busulfan | 1 | 7 | 0.001 |
| Clofarabine | 1 | 12 | 0.003 |
| Gemcitabine | 1 | 11 | 0.002 |
| Cytarabine | 1 | 18 | 0.006 |
| **Doxorubicin** | - | - | - |
| **Mesna** | - | - | - |
| **Dacarbazine** | - | - | - |
| **Ifosfamide** | - | - | - |

**Patient 4**

Diagnosis: Osteosarcoma

Therapeutics received: Methotrexate, cisplatin, doxorubicin, ifosfamide

| **Drug** | **Targets Hit** | **Potential Targets** | **P-value (hypergeometric test)** |
| --- | --- | --- | --- |
| Vismodegib | 1 | 3 | 0.001 |
| **Methotrexate** | 2 | 31 | 0.010 |
| Thalidomide | 1 | 6 | 0.003 |
| Decitabine | 1 | 7 | 0.004 |
| Topotecan | 2 | 44 | 0.025 |
| Vinblastine | 2 | 45 | 0.025 |
| Imatinib | 1 | 10 | 0.009 |
| Dexamethasone | 1 | 12 | 0.013 |
| Fulvestrant | 1 | 10 | 0.009 |
| Mitomycin C | 1 | 16 | 0.022 |
| Dactinomycin | 1 | 24 | 0.047 |
| **Cisplatin** | - | - | - |
| **Doxorubicin** | - | - | - |
| **Ifosfamide** | - | - | - |

**Patient 5**

Diagnosis: Synovial Sarcoma

Therapeutics received: Mesna, Doxorubicin, Ifosfamide, Dacarbazine, Methotrexate, Cisplatin

| **Drug** | **Targets Hit** | **Potential Targets** | **P-value (hypergeometric test)** |
| --- | --- | --- | --- |
| Thalidomide | 1 | 6 | <0.001 |
| Fulvestrant | 1 | 10 | 0.001 |
| Dexamethasone | 1 | 12 | 0.001 |
| Vorinostat | 1 | 28 | 0.007 |
| Crizotinib | 2 | 147 | 0.023 |
| Vincristine | 1 | 35 | 0.010 |
| Sorafenib | 1 | 42 | 0.015 |
| Epirubicin | 1 | 37 | 0.011 |
| Vinblastine | 1 | 45 | 0.017 |
| Topotecan | 1 | 44 | 0.016 |
| **Methotrexate** | 1 | 31 | 0.008 |
| **Mesna** | - | - | - |
| **Dacarbazine** | - | - | - |
| **Ifosfamide** | - | - | - |
| **Doxorubicin** |  |  |  |
| **Cisplatin** |  |  |  |

**Patient 6**

Diagnosis: Ewing’s Sarcoma

Therapeutics received: Vincristine, Doxorubicin, Cyclophosphamide, Dactinomycin, Ifosfamide, Etoposide

| **Drug** | **Targets Hit** | **Potential Targets** | **P-value (hypergeometric test)** |
| --- | --- | --- | --- |
| Temozolomide | 1 | 1 | <0.001 |
| Thalidomide | 1 | 6 | <0.001 |
| Dexamethasone | 1 | 12 | <0.001 |
| Crizotinib | 2 | 147 | 0.005 |
| Vorinostat | 1 | 28 | 0.003 |
| Pazopanib | 1 | 28 | 0.003 |
| Epirubicin | 1 | 37 | 0.005 |
| Bosutinib | 2 | 199 | 0.013 |
| Sorafenib | 1 | 42 | 0.006 |
| Vinblastine | 1 | 45 | 0.007 |
| Topotecan | 1 | 44 | 0.007 |
| **Doxorubicin** | 1 | 104 | 0.035 |
| Vandetanib | 1 | 117 | 0.044 |
| **Vincristine** | - | - | - |
| **Cyclophosphamide** | - | - | - |
| **Dactinomycin** | - | - | - |
| **Ifosfamide** | - | - | - |
| **Etoposide** | - | - | - |

**Patient 7**

Diagnosis: Undifferentiated Pleomorphic Sarcoma

Therapeutics received: Nil

| **Drug** | **Targets Hit** | **Potential Targets** | **P-value (hypergeometric test)** |
| --- | --- | --- | --- |
| Chlorambucil | 1 | 9 | <0.001 |
| Crizotinib | 5 | 147 | <0.001 |
| Carfilzomib | 1 | 24 | <0.001 |
| Dactinomycin | 2 | 24 | <0.001 |
| Mitomycin C | 1 | 16 | 0.001 |
| Bortezomib | 1 | 35 | 0.001 |
| Methotrexate | 2 | 31 | 0.001 |
| Vandetanib | 3 | 117 | 0.001 |
| Docetaxel | 1 | 22 | 0.002 |
| Thiotepa | 1 | 9 | 0.002 |
| Doxorubicin Hydrochloride | 1 | 104 | 0.003 |
| Decitabine | 1 | 7 | 0.003 |
| Vinblastine sulfate | 1 | 45 | 0.005 |
| Bexarotene | 1 | 13 | 0.006 |
| Nilotinib | 1 | 66 | 0.008 |
| Topotecan Hydrochloride | 1 | 44 | 0.008 |
| Sorafenib tosylate | 1 | 42 | 0.01 |
| Idarubicin hydrochloride | 1 | 44 | 0.012 |
| Vorinostat | 1 | 28 | 0.012 |
| Cytarabine | 1 | 18 | 0.012 |
| Axitinib | 5 | 105 | 0.013 |
| Carmustine | 1 | 9 | 0.013 |
| Paroxetine hydrochloride | 1 | 21 | 0.014 |
| Fluoruoracil | 1 | 8 | 0.014 |
| Clofarabine | 1 | 12 | 0.016 |
| Paclitaxel | 1 | 35 | 0.02 |
| Gefitinib | 1 | 88 | 0.023 |
| Bosutinib | 5 | 199 | 0.025 |
| Dasatinib | 1 | 145 | 0.044 |

**Patient 8**

Diagnosis: Alveolar Rhabdomyosarcoma

Therapeutics received: Vincristine, Cisplatin, Doxorubicin, Etoposide, Ifosfamide, Cyclophosphamide, Temozolomide

| **Drug** | **Targets Hit** | **Potential Targets** | **P-value (hypergeometric test)** |
| --- | --- | --- | --- |
| Letrozole | 2 | 5 | <0.01 |
| Methotrexate | 1 | 31 | 0.008 |
| **Vincristine sulfate** | **1** | **35** | **0.01** |

**Patient 9**

Diagnosis: Leiomyosarcoma

Therapeutics received: Nil

| **Drug** | **Targets Hit** | **Potential Targets** | **P-value (hypergeometric test)** |
| --- | --- | --- | --- |
| Belinostat | 1 | 9 | 0.002 |
| Etoposide | 1 | 24 | 0.011 |
| Pazopanib hydrochloride | 1 | 28 | 0.015 |
| Dactinomycin | 1 | 24 | 0.011 |
| Vorinostat | 1 | 28 | 0.015 |
| Epirubicin hydrochloride | 1 | 37 | 0.026 |

**Patient 10**

Diagnosis: Chordoma

Therapeutics received: Nil

| **Drug** | **Targets Hit** | **Potential Targets** | **P-value (hypergeometric test)** |
| --- | --- | --- | --- |
| Dexamethasone | 2 | 12 | <0.001 |
| Dactinomycin | 2 | 24 | <0.001 |
| Topotecan | 2 | 44 | 0.002 |
| Thalidomide | 1 | 6 | 0.001 |
| Cyclophosphamide | 1 | 7 | 0.001 |
| Busulfan | 1 | 7 | 0.001 |
| Carmustine | 1 | 9 | 0.001 |
| Mitomycin C | 1 | 16 | 0.004 |
| Cytarabine | 1 | 18 | 0.005 |
| Etoposide | 1 | 24 | 0.009 |
| Vorinostat | 1 | 28 | 0.012 |
| Methotrexate | 1 | 31 | 0.015 |
| Vincristine | 1 | 35 | 0.018 |
| Tamoxifen | 1 | 39 | 0.023 |
| Epirubicin | 1 | 37 | 0.02 |
| Sorafenib | 1 | 42 | 0.026 |
| Vinblastine | 1 | 45 | 0.03 |

**Patient 11**

Diagnosis: Metastatic Angiosarcoma

Therapeutics received: Nil

| **Drug** | **Targets Hit** | **Potential Targets** | **P-value (hypergeometric test)** |
| --- | --- | --- | --- |
| Fluorouracil | 1 | 8 | <0.01 |
| Fulvestrant | 1 | 10 | <0.01 |
| Chlorambucil | 1 | 9 | <0.01 |
| Carfilzomib | 1 | 24 | 0.002 |
| Vorinostat | 1 | 28 | 0.003 |
| Bortezomib | 1 | 35 | 0.004 |
| Vinblastine sulfate | 1 | 45 | 0.007 |
| Idarubicin Hydrochloride | 1 | 44 | 0.007 |
| Doxorubicin Hydrochloride | 1 | 104 | 0.035 |
| Axitinib | 1 | 105 | 0.036 |

**Patient 12**

Diagnosis: Undifferentiated Pleomorphic Sarcoma

Therapeutics received: Nil

| **Drug** | **Targets Hit** | **Potential Targets** | **P-value (hypergeometric test)** |
| --- | --- | --- | --- |
| Ruxolitinib | 1 | 2 | <0.001 |
| Fluorouracil | 1 | 8 | <0.001 |
| Fulvestrant | 1 | 10 | 0.001 |
| Trifluridine | 1 | 14 | 0.001 |
| Cytarabine | 1 | 18 | 0.002 |
